# Over-expression of the *Arabidopsis* proton-pyrophosphatase *AVP1* enhances transplant survival, root mass, and fruit development under limiting phosphorus conditions

**DOI:** 10.1093/jxb/eru149

**Published:** 2014-04-10

**Authors:** Haibing Yang, Xiao Zhang, Roberto A. Gaxiola, Guohua Xu, Wendy Ann Peer, Angus S. Murphy

**Affiliations:** ^1^Department of Horticulture, Purdue University, West Lafayette, IN 47907, USA; ^2^State Key Laboratory of Crop Genetics and Germplasm Enhancement, College of Resources and Environmental Sciences, Nanjing Agricultural University, Nanjing 210095, China; ^3^School of Life Sciences, Arizona State University, Tempe, AZ 85287, USA; ^4^Department of Environmental Science and Technology, University of Maryland, 1443 Animal Sciences, College Park, MD 20742, USA; ^5^Department of Plant Science and Landscape Architecture, University of Maryland, 2106 Plant Science Building, College Park, MD 20742, USA

**Keywords:** Fruit development, H^+^-pyrophosphatase, phosphorus, root development, tomato, transplant efficiency.

## Abstract

Phosphorus (P), an element required for plant growth, fruit set, fruit development, and fruit ripening, can be deficient or unavailable in agricultural soils. Previously, it was shown that over-expression of a proton-pyrophosphatase gene *AVP1/AVP1D* (*AVP1D*OX) in *Arabidopsis*, rice, and tomato resulted in the enhancement of root branching and overall mass with the result of increased mineral P acquisition. However, although *AVP1* over-expression also increased shoot biomass in *Arabidopsis*, this effect was not observed in tomato under phosphate-sufficient conditions. *AVP1D*OX tomato plants exhibited increased rootward auxin transport and root acidification compared with control plants. *AVP1D*OX tomato plants were analysed in detail under limiting P conditions in greenhouse and field trials. *AVP1D*OX plants produced 25% (*P*=0.001) more marketable ripened fruit per plant under P-deficient conditions compared with the controls. Further, under low phosphate conditions, *AVP1D*OX plants displayed increased phosphate transport from leaf (source) to fruit (sink) compared to controls. *AVP1D*OX plants also showed an 11% increase in transplant survival (*P*<0.01) in both greenhouse and field trials compared with the control plants. These results suggest that selection of tomato cultivars for increased proton pyrophosphatase gene expression could be useful when selecting for cultivars to be grown on marginal soils.

## Introduction

The essential macronutrient phosphorus (P) is relatively unavailable to plants in many soils when present as calcium salts or iron–aluminum oxide complexes (Holford, 1997). Tomatoes grown in soils with low phosphorus availability manifest deficiency symptoms such as stunting and leaf darkening before flowering and almost always exhibit reduced fruit size and production (Havlin *et al.*, 2005). For this reason, fertilization of tomato crops with P just before fruit set is a standard component of cultivation practices (Egel *et al.*, 2008).

Rising fertilizer costs and environmental damage resulting from phosphorus run-off have stimulated interest in strategies to enhance the availability of existing soil phosphorus. Inoculation of soils with mycorrhizal fungi and no-till agricultural practices have made a substantial contribution to the reduction of P-fertilizer applications (Bolan *et al.*, 1991). Tobacco plants engineered to overproduce the organic acid citrate have also been shown to enhance P uptake (López-Bucio, 2000). More recently, over-expression of the proton-pyrophosphatase in *Arabidopsis*, rice, tomato, and maize was reported to enhance P utilization (Yang *et al.*, 2007; Pei *et al.*, 2012). By contrast, the loss or the reduction of proton-pyrophosphatase *AVP1* function in *Arabidopsis* results in reduced shoot and root growth (Li *et al.*, 2005, Ferjani *et al.*, 2011).

AVP1 is a proton pump that utilizes the energy released by hydrolysis of a pyrophosphate (PP_i_) into two molecules of phosphate (P_i_) to acidify the vacuole. AVP1 is thought to function primarily in young, growing tissues where V-ATPase activity is reduced by depleted ATP reserves (Shiratake *et al.*, 1997; Zhen *et al.*, 1997; Maeshima, 2000; Heinonen, 2001). Although primarily localized to the tonoplast (Drozdowicz and Rea, 2001; Li *et al*, 2005), AVP1 was also localized to the plasma membrane (Langhans *et al.*, 2001; Alexandersson *et al.*, 2004; Paez-Valencia *et al.*, 2011) and over-expression of *AVP1* results in increased abundance and activity of H^+^-ATPase at the plasma membrane in *Arabidopsis* (Li *et al.*, 2005; Yang *et al.*, 2007; Undurraga *et al.*, 2012). The increased H^+^-ATPase abundance observed in *AVP1* over-expressors accounts for the increased apoplastic acidification observed in these lines. A direct result of this acidification is accelerated transport of the phytohormone auxin, increased lateral root branching, augmented exudation of organic acids, and enhanced P mobilization (Li *et al.*, 2005; Yang *et al.*, 2007). *AVP1*OX *Arabidopsis* and rice developed larger roots and shoots under both normal and low P_i_ conditions. Further, *Arabidopsis AVP1*OX plants displayed drought and salt tolerance (Gaxiola *et al.*, 2001). However, tomato plants over-expressing of *AVP1D*, the E229D gain-of-function mutant of *AVP1* that has increased PP_i_ hydrolysis and H^+^-translocation activity (Zhen *et al.*, 1997; Park *et al.*, 2005), developed shoots and fruits comparable to control plants under normal P_i_ conditions (Yang *et al.*, 2007). Further, the drought resistance observed in greenhouse trails (Park *et al.*, 2005) could not be repeated in the field trials and long-term drought resistance was not observed in *AVP1D*OX tomato plants (data not shown).

The differences observed in tomato plants prompted an analysis, in larger scale and more detail, of the response of these transgenic lines to P_i_ deficiency under greenhouse and field conditions. The analysis of *AVP1D*OX tomato performance in greenhouse and field trials under moderate P_i_-deficient conditions, similar to those encountered in Indiana farm practice as described by the *Midwest Vegetable Production Guide for Commercial Growers 2008* (Egel *et al.*, 2008), is reported here. In greenhouse trials, *AVP1D*OX plants displayed significantly increased shoot and root mass, and fruit production under low P_i_ conditions, but increased only in root mass under normal P_i_ conditions. In the field trials, the total fruit fresh weights of *AVP1D*OX plants were only slightly higher than those of control plants. However, compared with control plants, significantly (25%) more mature fruit per plant were harvested from *AVP1D*OX plants compared with control plants, which is consistent with its recently reported role in fruit development (Mohammed *et al.*, 2012). *AVP1D*OX plants displayed higher phosphate transport from leaf (source) to fruit (sink) compared with the controls under P_i_-deficient conditions. Further, *AVP1D*OX plants showed greater auxin transport and root acidification than control plants. Increased transplant survival was observed in all trials compared with the controls, suggesting that increased expression of native H^+^-pyrophosphatase genes may be an important marker for improved transplant survival.

## Materials and methods

### Plant material and culture conditions

The generation of *AVP1D*-over-expressing (*AVP1D*OX) and empty-vector control transgenic tomato plants (*Solanum lycopersicum*, cultivar Money Maker) was described in Park *et al.* (2005) and Yang *et al.* (2007). Three lines (*AVP1D-1, -2*, and *-3*) were used in the hydroponic experiments. Two lines, *AVP1D-1* and *AVP1D-2*, were used in the large greenhouse trials and showed very similar results. The *AVP1D-2* line was used for field trials.

#### Greenhouse trials with sterilized soil 

Trials were conducted in the Purdue University Department of Horticulture and Landscape Architecture Research Growth Facility. Seeds were sown in cell packs and germinated in a mist greenhouse for 3 weeks, then transplanted to 2-gallon pots filled with sterilized loam soil that had been extensively washed, twice with 2% HCl and five times with deionized water. Plants were placed in the greenhouse using a randomized block design, black plastic was used to cover the soil surface to simulate field conditions, and drip irrigation was used for watering. Natural light was supplemented to 14-h days using high pressure sodium lights (150 µmol m^–2^ s^–1^). Plants were fertilized every 2 weeks with P_i_-free medium containing 20mM 2-(*N*-morpholino) ethanesulphonic acid (pH 5.8), 5.0mM KNO_3_, 2.0mM MgSO_4_, 2.0mM Ca(NO_3_)_2_, 50 µM iron ethylenediaminetetraacetate, 70 µM H_3_BO_3_, 14 µM MnCl_2_, 0.5 µM CuSO_4_, 1.0 µM ZnSO_4_, 0.2 µM NaMoO_4_, 10 µM NaCl, and 0.01 µM CoCl_2_. The P_i_ concentration of medium was adjusted to 1mM with KH_2_PO_4_ for P_i_-sufficient conditions. For low-P_i_ treatments, 0.1% water-washed rock phosphate/phosphorite (Fisher) by weight was incorporated into the pot soil.

#### Field trials 

Experiments were conducted at the Throckmorton–Purdue Agricultural Center (TPAC) in south Tippecanoe County, Indiana. The farm has been extensively tiled for optimum drainage and set up for drip irrigation. Plants were watered and fertilized through the dripping system. A plot measuring 37×26 m was selected for tomato field trial experiments. Soils were sampled from random locations in the plot and tested for available macronutrient content by A&L Great Lakes Laboratories (Fort Wayne, IN). Very similar results were obtained from three sets of samples. The soil contained 22 µg g^–1^ P [Bray P1 method, medium for tomato according to Egel *et al.* (2008)], 3.3% organic matter, 117 µg g^–1^ potassium, 460 µg g^–1^ magnesium, 1900 µg g^–1^ calcium; the soil pH was 6.3 and buffer pH was 6.8. Tomato seeds were germinated and grown in cell packs for 30 d in the greenhouse. Seedlings were acclimated in a room without a roof close to the field for a week. With rows spaced 3 m apart and plants spaced 1 m apart in the row, 105 tagged control and *AVP1D-2* plants, respectively, were randomly transplanted into six beds covered with black plastic with 35 plants in each row. The test plants were surrounded by four border rows of untransformed wild-type tomato plants. Normal cultural practices for tomato cultivation, including drip irrigation, weed control, and pesticide treatments, were followed during the course of the experiment (Egel *et al.*, 2008). After 3 weeks of growth, tomato plants were supported with wires and stakes. No phosphate fertilizer was applied to the tested tomato plants during the experiments. Border plants were applied with phosphate-containing fertilizers through the drip irrigation system.

#### Greenhouse trials with unwashed soil of field trials 

Trials were conducted in the Purdue University Department of Horticulture and Landscape Architecture Research Growth Facility. Low-phosphate soil was obtained from the field of field trials (see above for soil test results) from Throckmorton–Purdue Agricultural Center (TPAC) in south Tippecanoe County, Indiana. Tomato seeds were germinated and grown in cell packs for 30 d in the greenhouse. Plants were randomly transplanted in 2-gallon pots on 64 trays, 1 control and 1 *AVP1D-2* in each tray. Plants were grown under P_i_-sufficient conditions for 1 month. Then 32 trays continued growth under P_i_-sufficient conditions and the other 32 trays were treated with low-P_i_. Plants were fertilized every 2 weeks with P_i_-free medium containing 20mM 2-(*N*-morpholino) ethanesulphonic acid (pH 5.8), 5.0mM KNO_3_, 2.0mM MgSO_4_, 2.0mM Ca(NO_3_)_2_, 50 µM iron ethylenediaminetetraacetate, 70 µM H_3_BO_3_, 14 µM MnCl_2_, 0.5 µM CuSO_4_, 1.0 µM ZnSO_4_, 0.2 µM NaMoO_4_, 10 µM NaCl, and 0.01 µM CoCl_2_. The P_i_ concentration of medium was adjusted to 1mM with KH_2_PO_4_ for P_i_-sufficient conditions. For low-P_i_ treatments, The P_i_ concentration of medium was adjusted to 10 µM with KH_2_PO_4_.

### Survival rate documentation

The living and dead plants were counted 1 week after transplantation into large pots for the greenhouse trial or soil for the field trial. Dead plants were not replaced to avoid the confounding effects of plants of different ages in subsequent analyses.

### Fresh weight determination

In the greenhouse experiments, control (vector alone) and *AVP1D*OX plants were harvested 100 d after transplantation. The shoot and fruit fresh weights were determined using a scale. The diameter of fruits was measured with calipers. The roots were carefully washed and blotted dry with paper towels before weights were determined with a scale. In the field trials, all control (90) and *AVP1D*OX plants (102) were harvested manually and dried in the bags. In order to minimize the moisture loss, shoot and fruit fresh weights for each plant were determined with a field scale immediately after harvesting the plants.

### Dry weight determination

In the greenhouse experiments, control and *AVP1D*OX plants were harvested 100 d after transplantation. The shoot and fruit were harvested. The roots were carefully washed and blotted dry with paper towels. Samples were dried in an oven 80 °C for 96h. Dry weights (DW) were determined using a balance.

### P transport assay

Stems (~15cm long) with 2–3 fruits and the closest compound leaf were cut from plants grown under normal and low-Pi conditions, and placed in a plastic container with 1mM and 10 µM Pi medium (above), respectively. Radiolabelled 20 µl 3.3 µM ^32^P-phospate solutions (1 µCi, 15 Ci mmol^–1^) were applied on the rachis of first two leaflets. The stem cuttings were placed under continuous light for 72h. The fruits were collected, weighed, and sliced into scintillation vials. 5ml scintillator liquid was added and the radioactivity was quantified by scintillation counting.

### Auxin transport assay

Auxin transport assays were performed as described in Liu *et al.* (2011).

### Root acidification assay

Plants were germinated in half-strength MS medium for 7 d, transferred to low-P_i_ medium as described above with 1mM MES, pH 6.8 and 0.04g l^–1^ bromocresol purple, and incubated for 3 d. The pH change was visualized via changes in medium colour. Comparisons were made with a colour bar generated by documenting the colour change of bromocresol purple in the same medium at specific pH values.

### Statistical analysis

Student’s *t* test and one-way analysis of variance (ANOVA), followed by pair-wise Holm–Sidak *post-hoc* analysis, was used to compare the development of roots, shoots, and fruits in *AVP1D*OX tomato versus control plants.

## Results

### Enhanced auxin transport and root acidification in *AVP1D*OX plants

**Fig. 1.**
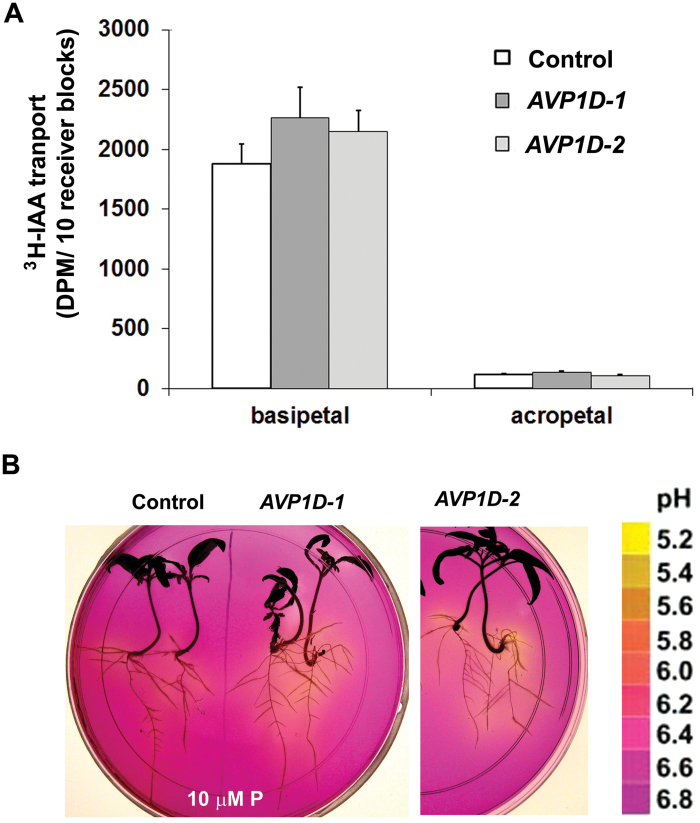
Enhanced basipetal auxin transport and root acidification in *AVP1D*OX plants. (A) Enhanced basipetal auxin transport in *AVP1D*OX plants. Values are means ±standard deviation (sd), *n*=3. (B) Pictures showed root acidification activity of tomato seedlings 3 d after transfer from control conditions to 10 µM P_i_ medium with the pH indicator bromocresol purple. (This figure is available in colour at *JXB* online.)

*AVP1*OX in *Arabidopsis* enhanced auxin transport and root acidification under low P_i_ conditions (Li *et al.*, 2005; Yang *et al.*, 2007). Similar results were observed in root acidification and auxin transport in *AVP1D*OX tomato seedlings (Fig. 1A). Enhanced auxin transport in *AVP1D*OX provided the explanation for larger roots measured in transgenic tomato plants. Further, *AVP1D-1* and *AVP1D-2* plants displayed increased acidification activity in medium under normal and low P_i_ conditions which also gave the basis for enhanced auxin transport (Fig. 1B). The function of *AVP1*OX in auxin transport and root acidification is conserved in *Arabidopsis* and tomato plants.

### Increased tolerance to P_i_ deficiency in transgenic tomatoes over-expressing *AVP1D* in greenhouse trials

**Fig. 2.**
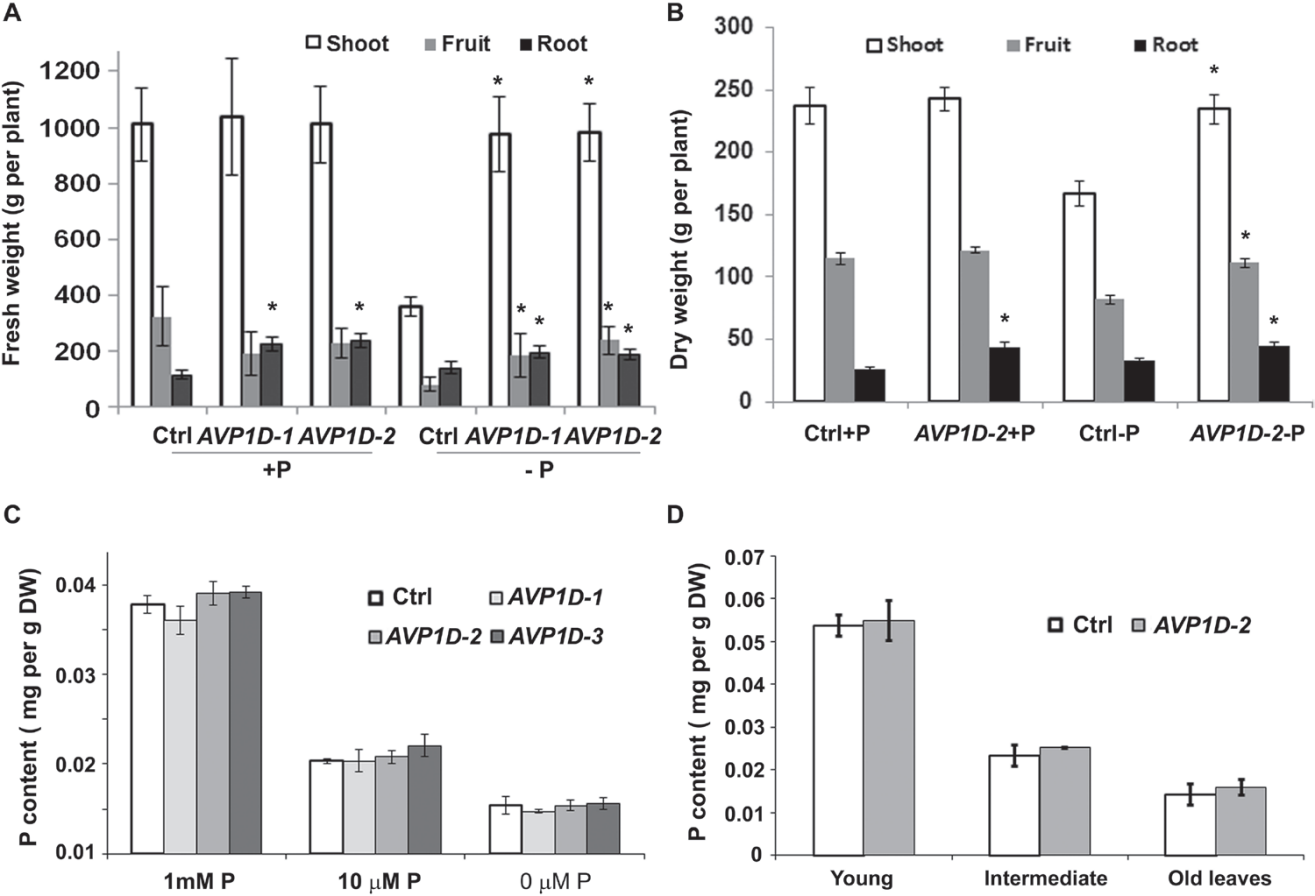
Performance of control and *AVP1D*OX plants under autoclaved loam soil, none-autoclaved low-P clay soil and hydroponic low-P conditions. (A) Greenhouse trials with washed and sterilized soil. The fresh weights of control and *AVP1D*OX plants under P-sufficient (+P, 1mM KH_2_PO_4_) and P-deficient (–P, 0.1% water-washed rock phosphate/phosphorite) conditions. Values are means ±standard deviation (sd), *n*=75. Asterisks indicate significance, ANOVA followed by Holm–Sidak *post-hoc* analysis, *P* <0.01, values from *AVP1D*OX plants were compared with values from control plants. (B) Greenhouse trials in untreated field soil. The dry weights of control and *AVP1D-2* plants under P-sufficient (+P, 1mM KH_2_PO_4_) and P-deficient (–P, 10 µM KH_2_PO_4_) conditions. Values are means ±standard deviation (sd), *n*=5. Asterisks indicate significance, Student’s *t* test, *P* <0.05, values from *AVP1D-2* plants were compared with values from control plants. (C) Average leaf P content in hydroponic control and *AVP1D*OX plants after 1 month treatment of 1mM, 10 µM, and 0 µM KH_2_PO_4_. Values are means ±standard deviation (sd), *n*=3. (D) P content in young, intermediate, and old leaves in hydroponic control and *AVP1D-2* plants after 1 month treatment of 1mM, 10 µM, and 0 µM KH_2_PO_4_. Values are mean ±standard deviation (sd), *n*=3.

Unlike *AVP1*OX *Arabidopsis* plants, *AVP1D*OX tomato plants did not show long-term drought tolerance or enhanced growth under normal P_i_ (400 ppm) soil conditions (Yang *et al.*, 2007). However, in this phenotypic analysis, a low P_i_ (0.5 ppm P) clay soil was used which has a high capacity to bind and immobilize P_i_. To eliminate the P_i_-sorption effect of soil, washed and sterilized loam soil was used to analyse the performance of *AVP1D*OX tomato plants under normal P_i_ (watered with 1mM P_i_ medium, see Materials and methods) and low P_i_ (rock phosphate) conditions. Two independent large greenhouse trials were performed each with 35 empty vector control plants, 35 *AVP1D-1* and 35 *AVP1D-2* plants. One hundred days after transplantation, fruits, shoots, and roots were harvested and weighed. Under P_i_-sufficient conditions (+P), a significant increase was seen in the root fresh weight of *AVP1D-1* and *AVP1D-2* compared with that of control plants (Fig. 2A, Student’s *t* test, *P* <0.01) but not in shoot mass and fruit yield. Under washed rock phosphate conditions (–P), control plants had reduced shoot and fruit biomass mass compared with +P controls (Fig. 2A) which was consistent with the switch from shoot growth to root growth to enhance P_i_ uptake under P_i_-limiting conditions. However, *AVP1D*OX plants produced comparable shoot, fruit, and root weights under washed rock phosphate conditions compared with +P plants (Fig. 2A), which was consistent with high acidification (Fig. 1A) and subsequent mobilization of P_i_ from rock phosphate, and also had increased root mass than the controls under the same conditions (Fig. 2A, *P* <0.05). Further, ~50% increase in fruit yield was observed in *AVP1D*OX plants compared with control plants under the rock phosphate conditions (Fig. 2A, P <0.01).

The performance of *AVP1D*OX plants was also tested using moderate low-P soil (22 µg g^–1^ P, Bray P1 method) from a field and watered with 1mM (+P) or 10 µM KH_2_PO_4_ (–P). The control and *AVP1D-2* plants were grown under normal or low P_i_ conditions for 3 months and the dry weights were compared between *AVP1D-2* and control plants. Similar to the fresh weight results in loam soil trials (Fig. 2A), *AVP1D-2* roots had the significantly greater dry weight (Fig. 2B, *P* <0.05), which indicates that *AVP1D*OX promotes root development under normal and low-P_i_ conditions. Greater shoot and fruit dry weights in *AVP1D-2* (Fig. 2B, *P* <0.05) supports the hypothesis that *AVP1D*OX increased P_i_ acquisition from high P-sorption clay soils, which was also consistent with higher rhizosphere acidification and a larger root system in *AVP1D-2* plants.

Since *AVP1D*OX increased shoot biomass and fruit weight compared with the control tomato plants in low-P conditions, it was hypothesized that *AVP1D*OX increased the P_i_ usage efficiency in plants through the translocation of P_i_ from older tissues to younger tissues. To test this hypothesis, control plants and three independent *AVP1D*OX transgenic lines *AVP1D-1*, *-2*, and *-3* were grown under hydroponic conditions in which P_i_ concentration and availability could easily be controlled. After treatment under the same low P_i_ conditions, no significant difference was seen in average leaf P content in *AVP1D*OX plants compared with the control plants (Fig. 2C). These results indicated that control plants can take up soluble P_i_ as efficiently as *AVP1D*OX plants under hydroponic conditions. Further, the P content in young, intermediate, and old leaves did not show a difference between *AVP1D-2* and control plants (Fig. 2D). These results indicate that *AVP1D*OX did not increase the P_i_ translocation (P_i_ usage efficiency) from old leaves to young leaves.

### Enhanced P_i_ translocation from leaves to fruits in *AVP1D*OX plants

**Fig. 3.**
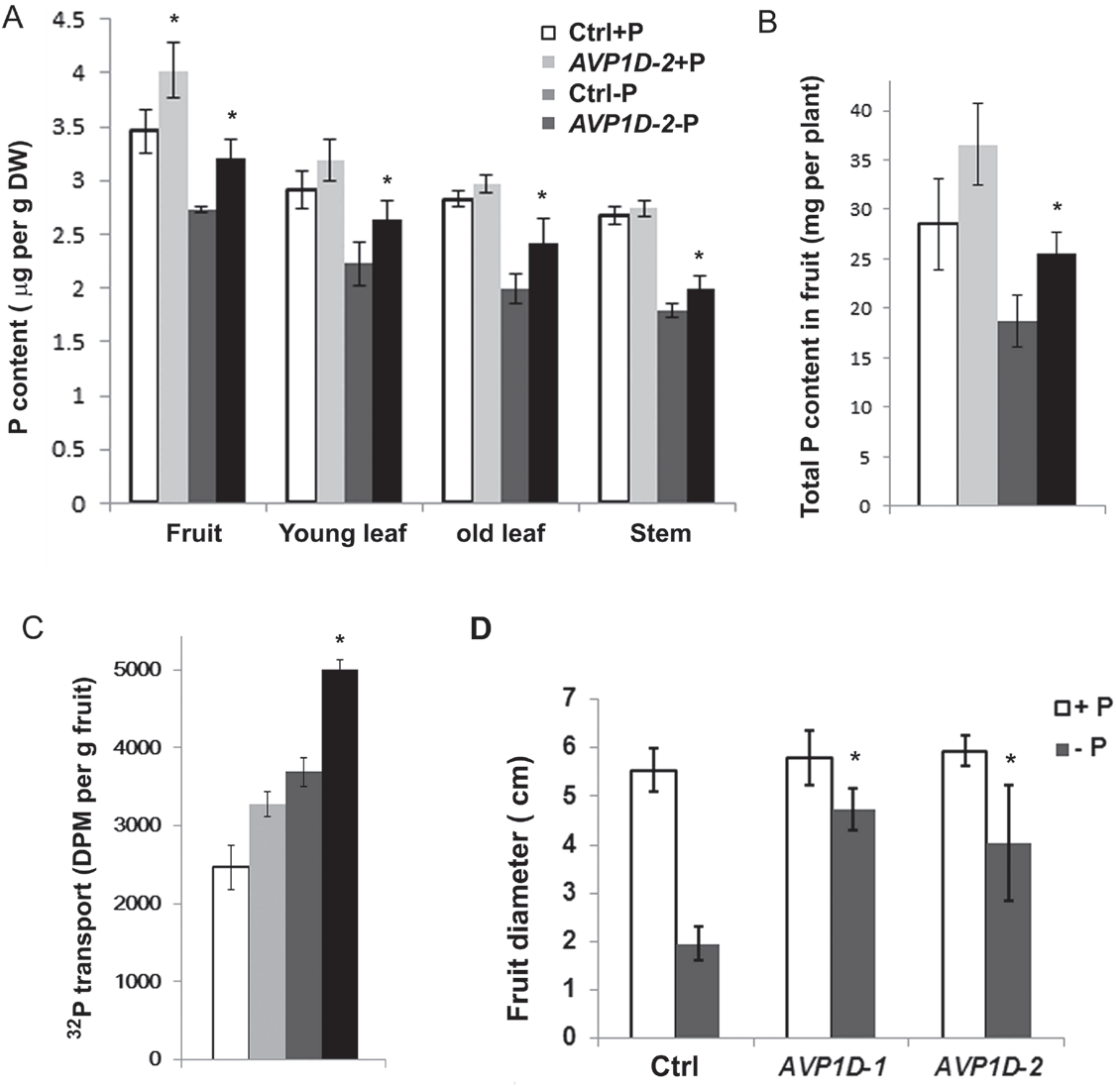
Enhanced P transport to fruits in *AVP1D-2*. (A) The P content of control and *AVP1D-2* plants under P-sufficient (+P) and P-deficient (–P) conditions. Values are means ±standard deviation (sd), *n*=4. Asterisks indicate significance, Student’s *t* test, *P* <0.05. (B) The total P content in fruits of control and *AVP1D-2* plants under P-sufficient (+P) and P-deficient (–P) conditions. Values are means ±standard deviation (sd), *n*=4. Asterisks indicate significance, Student’s *t* test, *P* <0.05. (C) Radiolabelled ^32^P-phospate transport from leave to fruits. Values are means ±standard deviation (sd), *n*=3. Asterisks indicate significance, Student’s *t* test, *P* <0.05. (D) Enhanced fruit development in *AVP1D*OX plants under P deficient conditions. Values are means ±standard deviation (sd), *n*=7. Asterisks indicate significance, Student’s *t* test, *P* <0.05.

To test the hypothesis that *AVP1D*OX enhances the P_i_ translocation to fruits, P contents in fruits were determined and P_i_ transport assays were performed using ^32^P-phosphate. Higher fruit P contents (µg g^–1^ dry weight) were measured in *AVP1D-2* compared with the controls under both normal and low P_i_ conditions (Fig. 3A, *P* <0.05). The P contents in *AVP1D-2* were also higher than the controls in leaves and stems when grown under low P_i_ soil conditions (Fig. 3A, *P* <0.05). Total P content in fruits (mg per plant) were higher in *AVP1D-2* than controls under low-P_i_ conditions (Fig. 3B, *P* <0.05). Enhanced transport of P_i_ detected in *AVP1D*OX stem cuttings, compared with the controls under low-P_i_ conditions, indicated the transport of ^32^P-phosphate from neighboring compound leaves into fruit (Fig. 3C, *P* <0.05). These data supported that *AVP1D*OX enhanced P_i_ translocation to fruits, which is consistent with the important role of type-I pyrophosphatases in tomato fruit development (Mohammed *et al.*, 2012). To confirm the role of *AVP1D*OX in fruit development further, the diameter of fruits was measured after 100 d growing in the greenhouse. *AVP1D-1* and *AVP1D-2* displayed enhanced fruit development compared with control plants under low-P conditions (Fig. 3D).

### Performance of *AVP1D*OX tomatoes grown without additional phosphorus in field trials

**Fig. 4.**
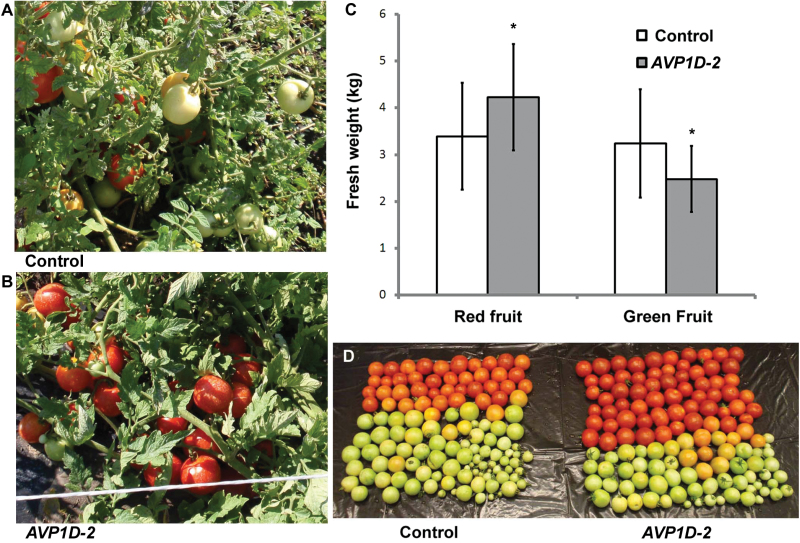
Field trials of control and *AVP1D*OX plants. Phosphate fertilizer was not applied during the course of the field trial. Field soil contained 22 µg g^–1^ P. (A) Control plant shows more green fruits. (B) *AVP1D*-2 plant shows more red fruits. (C) *AVP1D-2* plants produce significantly more red fruit fresh weight per plant than control plants. Values are means ±standard deviation (sd), *n*=85. Asterisks indicate significance, Student’s *t* test, *P* <0.01. (D) Total fruit from a representative control and an *AVP1D-2* plant, respectively.

The large greenhouse trial results were consistent with the results obtained in the small greenhouse trial with the low-P clay soil experiments (Yang *et al.*, 2007). However, plants may develop different root systems when grown under field conditions as space limitation is different in pots and field, and the response of control and *AVP1D*OX plants to low P_i_ may be different under field conditions. To test this possibility, the control and *AVP1D-2* plants were planted randomly in the field with 105 plants of each genotype distributed in six rows (see Supplementary Fig. S1A at *JXB* online). Shoots and fruits were harvested after 3 months in the fields (when >95% of the fruits were red in border plants with normal P_i_ conditions), and fresh weights were documented. Shoot fresh weight per plant of *AVP1D-2* plants was slightly greater than that of control plants (see Supplementary Fig. S1B at *JXB* online, *P*=0.06, Student’s *t* test). Both control and *AVP1D-2* plants showed low-P symptoms such as purple leaves after 3 months in field. The leaves and stems of control plants were more chlorotic than those of *AVP1D*OX plants (see Supplementary Fig. S1C–F at *JXB* online). These results were consistent with enhanced P_i_ acquisition by *AVP1D*OX in the clay soil greenhouse experiments (Fig. 2B, 3A). Fruit fresh weights of *AVP1*OX plants were not different from those of the control plants (see Supplementary Fig. S1B at *JXB* online, *P*=0.7). However, *AVP1D-2* plants produced more mature fruits that control plants (Fig. 4A, B), and 0.85kg more mature fruit per plant were harvested from *AVP1D*OX plants, 25% more than that harvested from control plants (Fig. 4C, *P* <0.01). The weight ratio of red fruits to green fruits was 1:1 for controls and 1.7:1 for *AVP1*OX plants, respectively. These results indicate that *AVP1D*OX enhanced fruit development under limiting P field conditions. These results were also consistent with enhanced P_i_ transport from leaves to fruit and fruit development under low-P conditions (Fig. 3C, D). A recent report showed that tomato vacuolar H^+^-pyrophosphatases play a role in early fruit development (Mohammed *et al.*, 2012). Knock-down of pyrophosphatase in tomato resulted in fruit growth retardation (Mohammed *et al.*, 2012), and fruit growth retardation was more obvious in control plants than *AVP1D-2* plants (Fig. 4D). Further, phosphate is required for phosphate esterification during fruit ripening (Marks *et al.*, 1957). Our results suggest that *AVP1D*OX plants can transport more phosphate for fruit development and maturation under limiting phosphate conditions.

### Increased transplant survival rates of *AVP1D*OX transgenic plant in large-scale greenhouse and field trials

**Fig. 5.**
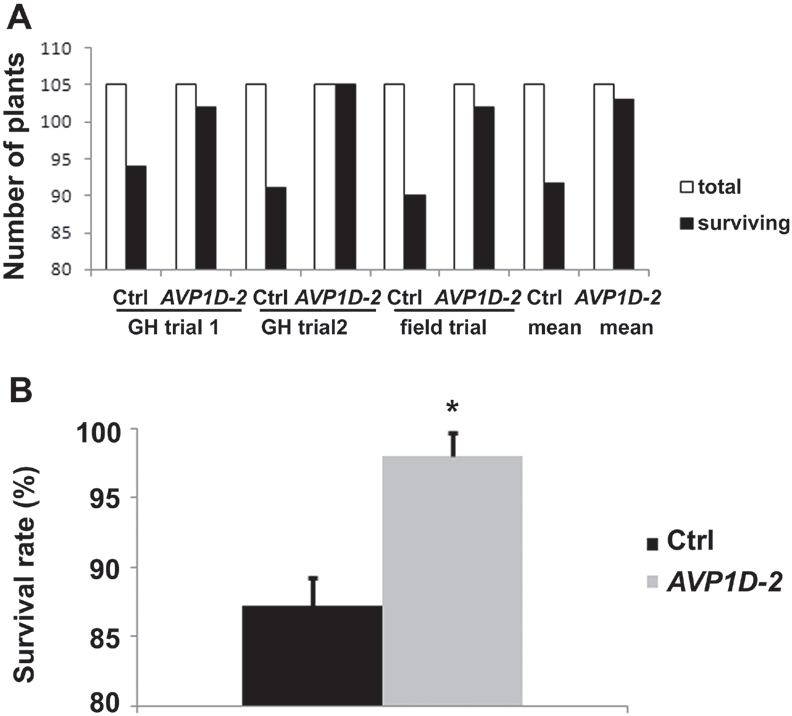
Survival rates of control and *AVP1*OX plants after transplantation. (A) Number of living plants after transplantation in large-scale greenhouse (GH) and field trails as indicated. Values are number of individuals. (B) Total transplant survival rate for both greenhouse and field trials. Values are means ±standard deviation (sd), *n*=3 trials. Asterisk indicates significance, Student’s *t* test, *P* <0.01.

Interestingly, transplant survival rate of *AVP1D*OX plants was higher than control plants in both large-scale greenhouse and field trials. 94 and 91 out of 105 (89% and 86%, respectively) control plants survived transplantation to large pots, while 102 (97%) and 105 (100%) of the 105 *AVP1*OX plants survived transplantation to large pots, respectively, in two large-scale greenhouse trials (Fig. 3A). In the field trials, 92 out of 105 control plants (87%) and 102 out of 105 *AVP1*OX plants (97%) survived transplantation to the field (Fig. 5A). Overall, transplant-survival rate for *AVP1*OX plants was 98%, which is significantly higher than the 87% transplant-survival rate in control tomato plants (Fig. 5B, *P*=0.002). Subsequent transplantation experiments performed in the greenhouse and in the field confirmed that those survival rates are consistently observed (data not shown). Although under our conditions control plants displayed decreased transplantation survival rates than expected, these conditions revealed differences between control and *AVP1D*OX plants. These results suggest that *AVP1* expression can be used as a selective marker in breeding efforts to identify lines with enhanced P utilization capacity and improved transplantation efficiency. The approach of *AVP1*OX can be applied to improve transplantation success for crops or trees with low transplantation efficiency.

## Discussion

### *AVP1* enhancement of plant performance in low-P soils: biomass versus fruit production

In both the large-scale greenhouse and the field trials, *AVP1D*OX tomato plants developed more biomass under P-deficient conditions than control plants. However, this biomass increase did not translate into fruit quantity, but fruit quality was enhanced as more mature fruit were developed in *AVP1D*OX plants (Fig. 4). In other words, *AVP1D*OX plants under low P produced the same amount of mature fruit as the wild type under P-sufficient conditions. *AVP1* over-expression has been shown to increase overall biomass accumulation and seed production by *Arabidopsis*, rice, and maize under both P-sufficient and P-deficient laboratory conditions (Yang *et al.*, 2007; Pei *et al.*, 2012). However, biomass and fruit production were not significantly increased in *AVP1*OX tomato plants under P-sufficient conditions, presumably due to species-specific developmental factors (reviewed in McSteen and Leyser, 2005). A recent study showed the localization of all three tomato pyrophosphatases to fruits and showed that their activity was critical for fruit development (Mohammed *et al.*, 2012) which supports this hypothesis. The diversification of pyrophosphatase in fruit plants suggests that tissue- or organ-specific *AVP1* over-expression may be valuable for enhancement of specific traits.

### *AVP1D*OX enhances fruit growth and ripening in tomatoes under P-deficient conditions

In field trials, *AVP1D*OX plants produced more mature fruits both in number and weight than control tomato plants without P supplementation (Fig. 4E, F). Fruit development can be divided into distinct periods: (1) cell division, (2) cell enlargement, (3) maturation, (4) ‘autogenous climacteric’, and (5) senescence. The climacteric stage is characterized by a sudden, sharp increase in respiratory rate and ethylene production in fruit ripening of climacteric fruits such as tomatoes, apples, bananas, melons, and apricots. Phosphate is required for fruit growth and phosphate esterification during fruit ripening (Marks *et al.*, 1957) and is consistent with the observation here that P deficiency resulted in more small, green tomatoes in control plants. Although other nutrients are also important for fruit development, P is critical for fruit set and growth, and P fertilizer application is common practice prior to fruit set in tomato fields. Our data indicated that *AVP1* plays an important role in the translocation of P for tomato fruit development. However, over-expression of *AVP1* in *Lactuca sativa* also enhances nitrogen use efficiency in leaves (Paez-Valencia *et al.*, 2013).

### *AVP1D* over-expression enhances root growth and transplantation survival

*AVP1D*OX tomato plants had higher transplantation survival rates (Fig. 5). Similar results were seen for *AVP1*OX rice plants under semi-dry conditions (data not shown). However, enhanced transplantation survival could be the result of enhanced root growth in *AVP1D*OX tomato plants as indicated by root weights (Fig. 2). Plants encounter multiple stresses during transplantation including root loss, wilting, wounding, drought, nutrition, disease, and other stresses (reviewed in Koller, 1977). Transplantation success rate is an important component of production methods, and cultivars with robust root systems are more likely to survive transplantation (Harris and Bassuk, 1993). *AVP1*OX in many species, *Arabidopsis*, alfalfa, rice, tomato, barley, maize, tobacco, and cotton developed larger root systems and are more resistant to salt and drought stress, and/or limiting P_i_ conditions (Gaxiola *et al.*, 2001; Park *et al.*, 2005; Zhao *et al.*, 2006; Bao *et al*., 2008; Li *et al.*, 2008; Lv *et al*., 2008, 2009; Pasapula *et al.*, 2010; Pei *et al.*, 2012; Schilling *et al.*, 2013). Enhanced short-term drought tolerance in *AVP1D*OX plants was consistent with higher transplantation survival rates (Park *et al.*, 2005). Increased auxin fluxes in *AVP1D*OX plants (Fig. 1) may also facilitate adventitious root initiation during transplantation. Increased auxin transport and root acidification were also observed in *Arabidopsis AVP1*OX plants which indicated that *AVP1*OX share similar roles in root development in *Arabidopsis* and tomato plants. However, the unique shoot and fruit phenotype in *AVP1D*OX tomato plants suggested that *AVP1D* plays a specific role in the transport of P_i_ between leaves (source) and fruits (sink tissue).

## Supplementary data

Supplementary data can be found at *JXB* online

Supplementary Fig. S1. Field trials of control and *AVP1*OX plants.

Supplementary Data
