# Supplementary material for: Over-expression of the Arabidopsis proton-pyrophosphatase AVP1 enhances transplant survival, root mass, and fruit development under limiting phosphorus conditions
Source: J Exp Bot. 2014 Apr 10;65(12):3045–53. doi: 10.1093/jxb/eru149 (PMC4071825; doi:10.1093/jxb/eru149)
Supplement: Supplementary Data [file supp_65_12_3045__index.html]

Over-expression of the Arabidopsis proton-pyrophosphatase AVP1 enhances transplant survival, root mass, and fruit development under limiting phosphorus conditions — Over-expression of the Arabidopsis proton-pyrophosphatase AVP1 enhances transplant survival, root mass, and fruit development under limiting phosphorus conditions — Supplementary Data 

# Over-expression of the *Arabidopsis* proton-pyrophosphatase *AVP1* enhances transplant survival, root mass, and fruit development under limiting phosphorus conditions

## Supplementary Data

Data files

**Files in this Data Supplement:**

- Supplementary Data - Supplementary Data
