# Supplementary material for: Over-expression of the Arabidopsis proton-pyrophosphatase AVP1 enhances transplant survival, root mass, and fruit development under limiting phosphorus conditions
Source: J Exp Bot. 2014 Apr 10;65(12):3045–53. doi: 10.1093/jxb/eru149 (PMC4071825; doi:10.1093/jxb/eru149)
Supplement: Supplementary Data [file supp_eru149_jexbot116038_file001.pdf]

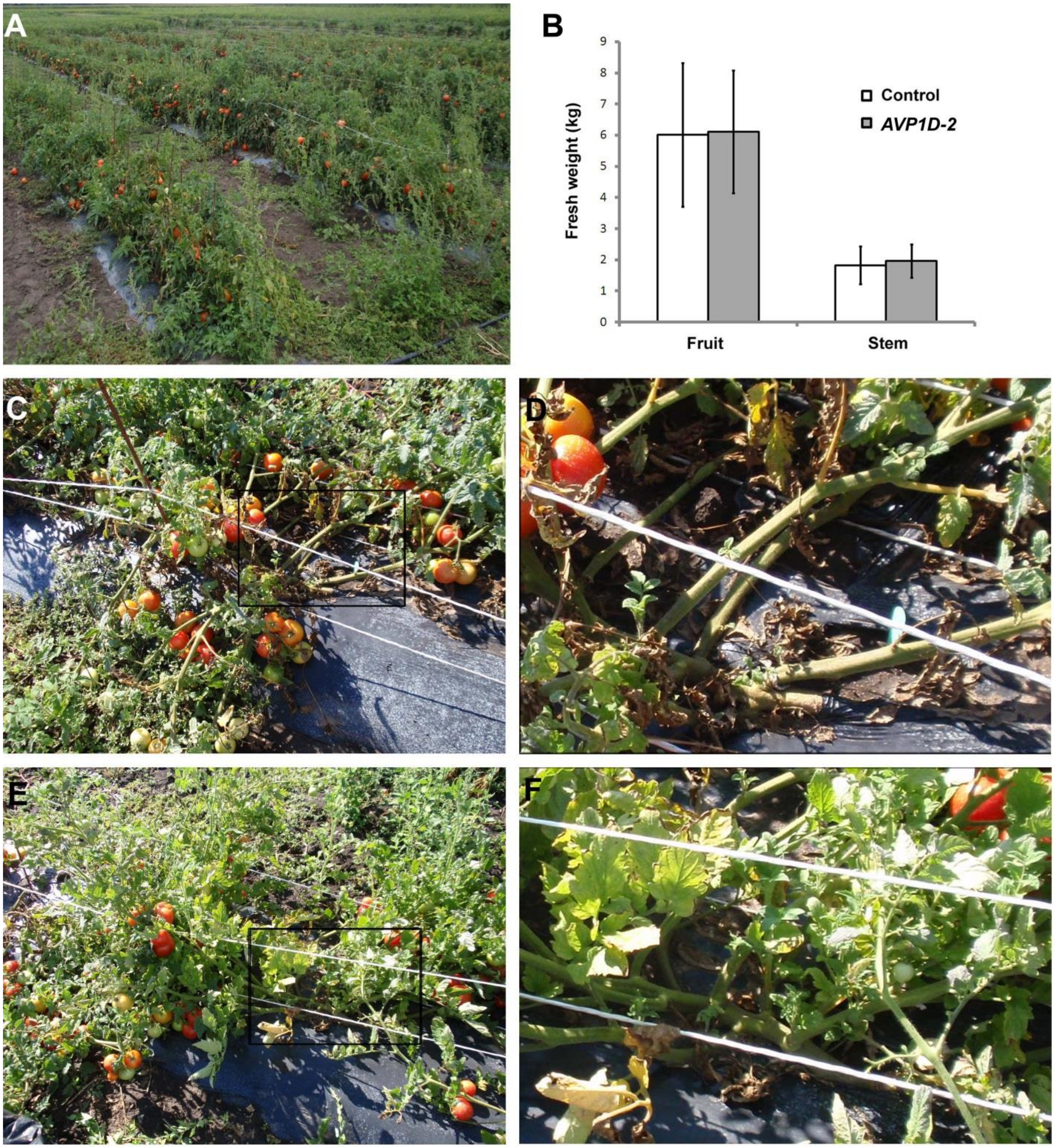

Figure S1. Field trials of control and *AVP1D*OX plants.

(A) Tomato field prior to harvesting. **(B)** The total fruit fresh weights of *AVP1D*OX plants were only slightly higher than those of control plants. The difference seen was not significant in total stem fresh weight per plant between control and *AVP1D-2* plants (P=0.06, Student's *t*-test). Values are means ± standard deviations, n = 85. **(C, D)** Control plant showed more chlorotic leaves (D, magnification). **(E,F)** *AVP1D-2* plant showed greener leaves (E, magnification).
